# Lens nucleus dislocation in hypermature cataract: Case report and literature review

**DOI:** 10.1097/MD.0000000000030428

**Published:** 2022-09-02

**Authors:** Jie-Ying Guan, Yun-Cheng Ma, Ying-Ting Zhu, Ling-Ling Xie, Mireayi Aizezi, Ye-Hong Zhuo, Aizezi Wumaier

**Affiliations:** a Kashgar Hospital Affiliated to Sun Yat-sen University, Xinjiang, China; b State Key Laboratory of Ophthalmology, Zhongshan Ophthalmic Center, Sun Yat-sen University, Guangdong Provincial Key Laboratory of Ophthalmology and Visual Science, Guangdong Provincial Clinical Research Center for Ocular Diseases, Guangzhou, China; c Department of Ophthalmology, the First People’s Hospital of Kashi, Xinjiang, China.

**Keywords:** case report, ectopia lentis, intracapsular cataract extraction, literature review, Morgagnian cataract

## Abstract

Hypermature cataract is a form of late-stage cataract progression that can lead to a variety of complications. Spontaneous capsular rupture with lens nucleus displacement in hypermature cataracts has rarely been reported. We describe 2 cases of spontaneous dislocation of the lens nucleus in a hypermature cataract and perform a review of the literature on this complication.

**Patient Concerns:**

We report 2 rural men aged 50 and 76 years with deteriorating vision.

**Diagnosis:**

The final diagnosis was senile hypermature cataract with dislocation of the lens nucleus in both patients and secondary glaucoma for the second patient.

**Interventions and Outcomes:**

During admission, both patients complained of deteriorating vision. Slit-lamp examination showed lens nucleus dislocation into the anterior chamber. The 50-year-old patient exhibited a residual lens capsule and a turbid cortex, with a normal anterior chamber and intraocular pressure. The 76-year-old patient presented a shrunken and ruptured capsule and no cortex in the pupillary area, mild inflammation in the anterior chamber, and high intraocular pressure. Both patients underwent intracapsular cataract extraction combined with anterior vitrectomy and achieved good postoperative recovery.

**Conclusion:**

Lens nucleus dislocation in hypermature cataracts can be seen in clinical practice, particularly in underdeveloped areas. Early recognition and surgery can improve vision.

## 1. Introduction

With improvements in the standards of living and health awareness, many cataract patients have been cured by surgical procedures in the early and middle stages of the disease. However, in some remote and less developed areas, hypermature cataracts continue to be a problem. Hypermature cataracts can lead to many complications, including lens-induced uveitis,^[1]^ secondary glaucoma,^[2]^ and lens or lens nucleus dislocation.^[3,4]^ However, spontaneous capsular rupture with lens nucleus displacement has rarely been reported. Herein, we describe 2 cases of spontaneous dislocation of the lens nucleus in a hypermature cataract. We review the literature on lens nucleus dislocation focusing on the diagnosis and treatment of hypermature cataracts in these specific cases.

## 2. Case presentation

**Figure 1.**
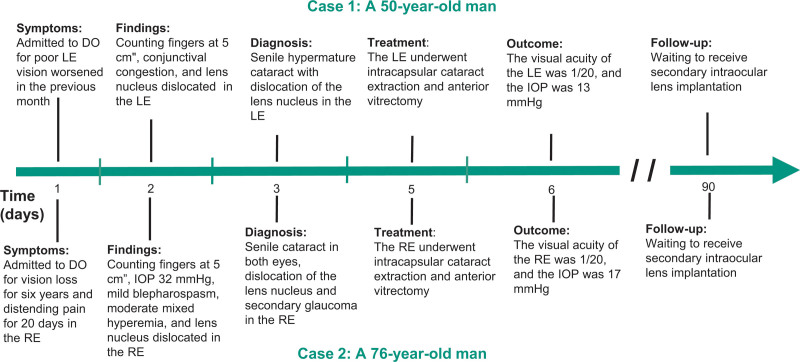
Clinical timeline. DO = Department of Ophthalmology, IOP = intraocular pressure, LE = left eye, RE = right eye.

This study adhered to the tenets of the Declaration of Helsinki and was approved by the Human Research Ethics Committee of the First People’s Hospital of Kashi (ethics no. 2021ksyd-18). Both participants provided written informed consent. The clinical timeline of the cases is shown in Figure 1.

### 2.1. Case 1

A 50-year-old man from a rural area was admitted to the Department of Ophthalmology of the First People’s Hospital of Kashi due to poor left eye (LE) vision that had been deteriorating over the last 12 years, with recent worsening of symptoms during the previous month. No eye pain or redness was noted upon admission. The patient reported “gastric repair” for “gastric perforation” 3 years prior but denied having a history of eye trauma or other diseases. Otherwise, personal and family histories contained no relevant details.

**Figure 2.**
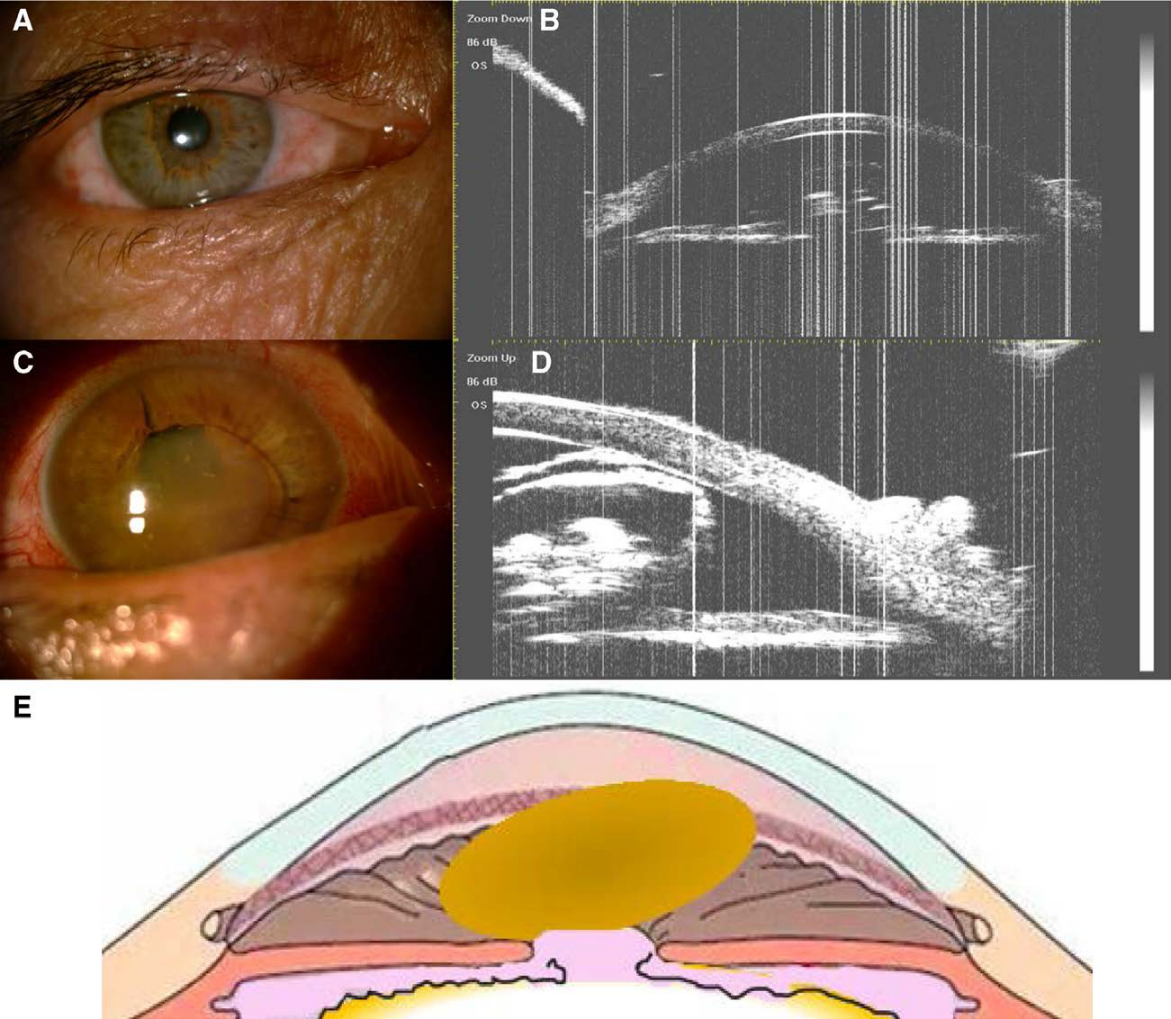
Slit-lamp (×16 magnification) and UBM (×40 magnification) images and a representative illustration of the hypermature cataract in case 1. (A) Frontal view of the entire RE. (B) Frontal view of the entire LE, which shows the pale brown, disc-shaped nucleus dislocated into the anterior chamber. Crystal particles can also be observed. (C, D) UBM images of the LE show the lens nucleus dislocation in the anterior chamber. (E) Lens nucleus dislocation to the anterior chamber in this case of hypermature cataract. LE = left eye, RE = right eye, UBM = ultrasound biomicroscopy.

On examination, the best corrected visual acuity was 20/20 in the right eye (RE), and counting fingers at 5 cm in the LE with a normal visual field and red-green color perception. The intraocular pressures (IOPs) measured with Goldmann applanation tonometry were 12 mm Hg and 8 mm Hg in the RE and LE, respectively. Slit-lamp examination showed no abnormalities of the RE (Fig. 2A). The LE revealed conjunctival congestion and a clear cornea (Fig. 2B). The lens nucleus was pale brown, disc-shaped, and dislocated into the anterior chamber. Because of the medium-sized pupil, the lens could not return to the posterior chamber. A residual lens capsule and turbid cortex were observed in the pupillary area. The anterior chambers were normal, and no signs of uveitis were noted. Ultrasound biomicroscopy showed that the depth of the central anterior chamber was 2.22 mm in the RE and 3.26 mm in the LE (Fig. 2C, D). A representative illustration of the dislocated nucleus, in this case, is shown in Figure 2E. Corneal endothelioscopy showed that the cell density of the LE was 2910.2/mm^2^ with 38% hexagonal cells. Optical coherence tomography (OCT) (Spectralis OCT; Heidelberg Engineering, Heidelberg, Germany) and B-scan ultrasonography (Resona 7; Mindray, Shenzhen, China) revealed a flat retina in the LE. All laboratory examinations were within normal ranges.

The patient was diagnosed with a senile hypermature cataract with dislocation of the lens nucleus in the LE. He was subjected to intracapsular cataract extraction and anterior vitrectomy due to a rupture of the capsular bag. Postoperative recovery was excellent. Visual acuity and IOP of the operative eye were 1/20 and 13 mm Hg, respectively, on postoperative day 1, with no signs of cells or flares in the anterior chamber. No unusual discomfort was reported. At this point, the patient was waiting for secondary intraocular lens implantation.

### 2.2. Case 2

A 76-year-old man from rural presented with a history of vision loss during the prior 6 years and mild-to-moderate distending pain during the last 20 days in the RE. He was admitted to the same hospital. The RE was slightly reddish upon admission. No nausea or vomiting was noted. The patient was referred to be healthy in the past and denied a history of trauma, systemic symptoms, or disorders. Nothing of relevance was reported regarding personal or familial medical history.

**Figure 3.**
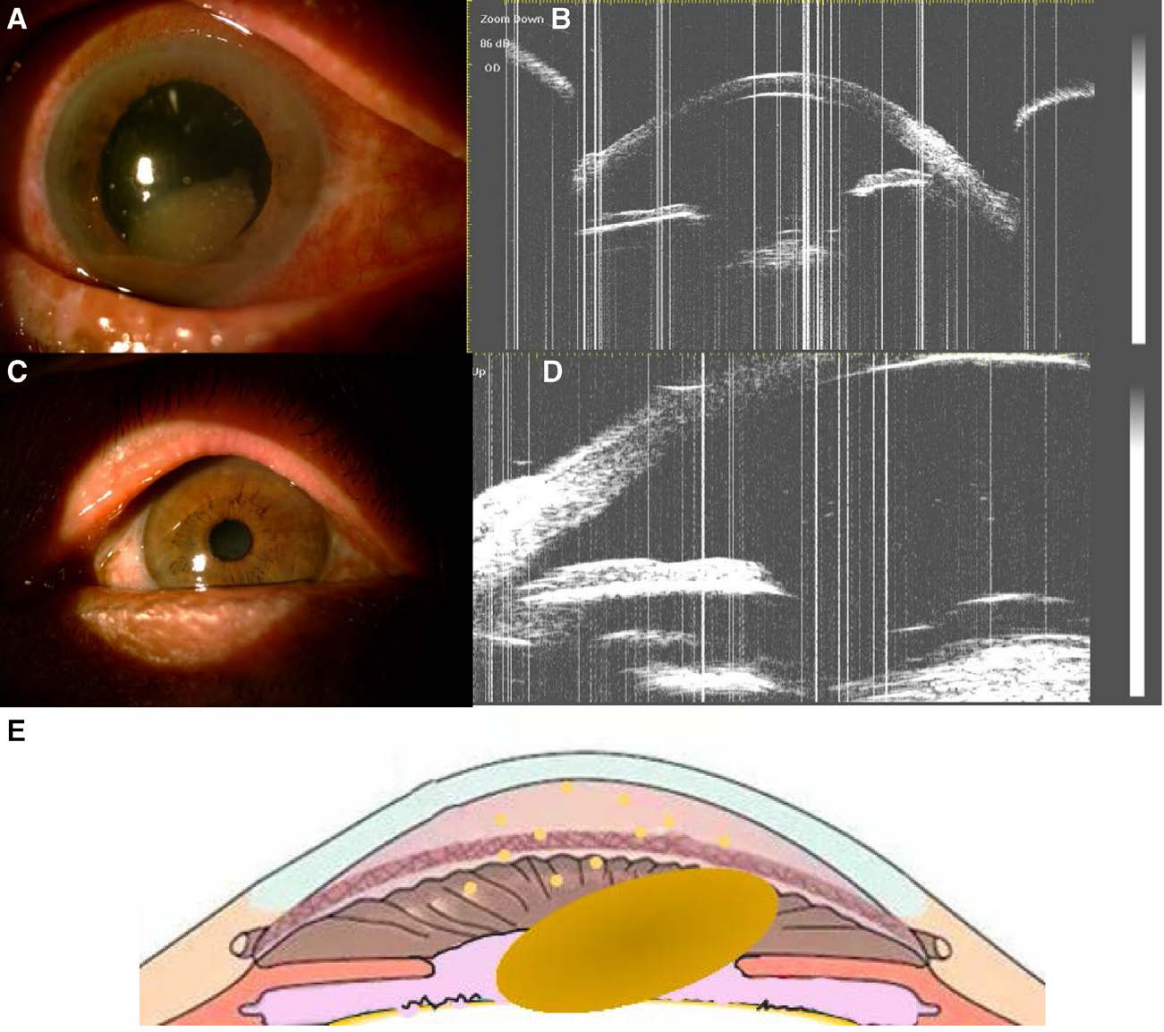
Slit-lamp (×16 magnification) and UBM (×40 magnification) images and a representative illustration of the hypermature cataract in case 2. (A) Frontal view of the entire RE, which exhibits a pale brown, disc-shaped nucleus dislocated into the anterior chamber. The shrunken and ruptured capsule can be seen in the pupillary area with no cortex. (B) Frontal view of the entire LE. (C, D) UBM images of the RE show the lens nucleus dislocation in the posterior chamber due to the patient’s supine position. We also observed granular reflections in the anterior chamber. (E) Lens nucleus dislocation to the anterior chamber in this case of hypermature cataract. LE = left eye, RE = right eye, UBM = ultrasound biomicroscopy.

On examination, the best corrected visual acuity was counting fingers at 5 cm in the RE with a normal visual field and red-green color perception, and 5/20 in the LE. The IOPs were 32 mm Hg and 20 mm Hg in the RE and LE, respectively. Slit-lamp examination of the RE revealed mild blepharospasm, moderate mixed hyperemia, and a clear cornea. The lens nucleus was pale brown, disc-shaped, and dislocated into the anterior chamber. Because of the dilated and fixed pupil, the lens could return to the posterior chamber and move with the body position. There was a shrunken and ruptured capsule and no cortex in the pupillary area. Mild inflammation in the anterior chamber was observed in the RE (Fig. 3A). The lens cortex was slightly gray and white in the LE (Fig. 3B). Ultrasound biomicroscopy showed that the depth of the central anterior chamber was 3.14 mm in the RE and 2.09 mm in the LE (Fig. 3C, D). A sketch map of this case is shown in Figure 3E. Corneal endothelioscopy revealed that the cell density of the RE was 2431.6/mm^2^ with 33% hexagonal cells. The OCT and B-scan ultrasonography images showed a flat retina in the RE. All laboratory examinations were within normal ranges.

The patient was diagnosed with hypermature and primary stage cataracts in the RE and LE, respectively, with dislocation of the lens nucleus and secondary glaucoma in the RE. Intracapsular cataract extraction and anterior vitrectomy due to rupture of the capsular bag were the chosen course of action. Postoperative recovery was excellent. Visual acuity and IOP of the operative eye were 1/20 and 17 mm Hg, respectively, on postoperative day 1, with no signs of cells or flares in the anterior chamber. No unusual discomfort was reported. At this point, the patient was waiting for secondary intraocular lens implantation.

## 3. Discussion

**Figure 4.**
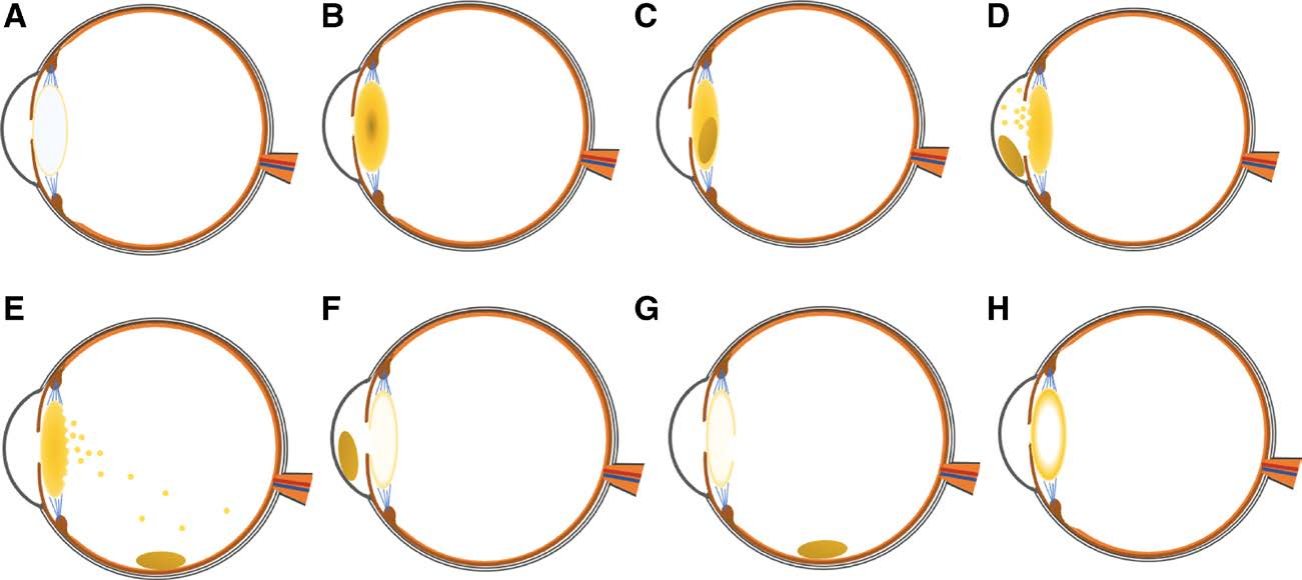
Schematic diagrams of hypermature cataracts. (A) A clear lens. (B) A nuclear cataract. (C) A cortical or Morgagnian cataract. (D) A hypermature cataract with increased anterior capsule permeability or rupture, cortical overflow, and lens nucleus detachment into the anterior chamber. (E) A hypermature cataract with increased posterior capsule permeability or rupture, cortical overflow, and lens nucleus detachment into the vitreous cavity. (F) A hypermature cataract with spontaneous absorption of the lens cortex and lens nucleus detachment into the anterior chamber from the capsule rupture hole. (G) A hypermature cataract with spontaneous absorption of the lens cortex and lens nucleus detachment into the vitreous cavity from the capsule rupture hole. (H) A hypermature cataract with spontaneous absorption of the lens nucleus and residual lens capsule bag and cortex.

Hypermature cataracts are a manifestation of late cataract progression. As a cortical cataract, also called “Morgagnian cataract,” progresses, the following changes occur: water is lost, the lens volume decreases, the capsule shrinks, calcification or cholesterol crystals appear on the lens surface, lens fibers decompose and liquefy into milky white particles, and the solid nucleus sinks to the bottom.^[3]^ Degeneration of the capsule or the impact of the lens nucleus can cause an increase in the permeability of the capsule or cause it to rupture. This can result in the liquefied lens cortex overflowing into the anterior chamber, which in turn can lead to lens-induced uveitis or phacolytic glaucoma. The lens is prone to dislocation or displacement because of the degeneration of the lens suspensory ligament. Rupture of the capsule can also cause nuclear prolapse.^[1,2]^ The specific mechanism of this condition is illustrated in Figure 4.

In the past 10 years, there have been few reports of nucleus dislocation in hypermature cataracts. We have summarized the available case reports in Table 1.^[5–11]^ This may be related to our understanding of cataracts and the improvements in ophthalmic examination and surgical techniques. In these cases, we observed the lens nucleus sinking into the capsular bag and spontaneously dislocating into the anterior chamber or vitreous cavity. Complications such as capsular bag calcification, uveitis, elevated IOP, macular edema, and macular hole were seen in some previously reported cases. Goel and Nagar^[4]^ analyzed the clinical data of ten cases of spontaneous rupture of the lens capsule in hypermature cataracts. Among these cases, 3 eyes exhibited anterior dislocation, 2 eyes exhibited posterior dislocation, and the other 5 eyes exhibited absorption of the lens nucleus. Tearing of the anterior or posterior capsule was observed in 8 of 10 eyes, and cystic calcification was found in only 5 eyes. IOP was increased in 1 eye, decreased in 1 eye, and normal in the remaining 8 eyes.

**Table 1:** Literature on lens nucleus dislocation in hypermature cataracts.

Author	Year of publication	Age of patient (yr)	Inflammation of anterior chamber	Intraocular pressure	Clinical features
Gupta et al^[5]^	2020	70	Normal	Normal	Nucleus sinking into the capsular bag and capsule calcification
Deshmukh et al^[6]^	2020	56	Normal	Normal	Morgagnian cataract in the right eye and lens absorption in the left eye
Petrovic et al^[7]^	2020	79	Normal	High	Nucleus dislocated in the vitreous, development of cystoid macular edema with lamellar macular hole
Eadie et al^[8]^	2019	70	Mild	High	Nucleus sinking into the capsular bag
Dhingra et al^[9]^	2019	75	Normal	Normal	Nucleus dislocated in the anterior chamber, capsule calcification, and cortical absorption
Hemalatha et al^[10]^	2012	52	Normal	Normal	Nucleus dislocated in the anterior chamber; capsule calcification
Malik et al^[11]^	2014	50	Normal	Normal	Nucleus dislocated in the posterior chamber; capsule calcification

Herein, we report 2 cases of spontaneous rupture of the lens capsule with anterior dislocation of the lens nucleus caused by hypermature cataracts. In case 1, the dislocated lens nucleus was restricted to the anterior chamber due to a small pupil but did not result in lens-induced uveitis. The IOP was low, and the residual lens cortex could be seen by mydriasis before surgery. In case 2, the pupil was dilated, and no residual lens cortex was observed. The dislocated lens nucleus moved through postural changes and was accompanied by mild anterior chamber inflammation and elevated IOP. Based on previous literature,^[4–11]^ we hypothesized that the hypermature cataract causes the capsule to shrink and degenerate to form a tiny rupture hole. The lens cortex then either leaks into the vitreous cavity from the posterior capsule fissure and is gradually absorbed, or slowly decomposes and is absorbed in situ, preventing uveitis from occurring. Because the vitreous body was transparent as seen during anterior vitrectomy in case 2, we deduced the possibility of lens cortex absorption in situ. The nucleus suspended in the capsule can break through the fragile capsule and dislocate to the anterior chamber or vitreous body. White calcified spots on the capsule may indicate a spontaneous rupture of the lens capsule; however, we did not observe obvious calcified spots in either patient.

It should be noted that case 1 was a 50-year-old man with no history of trauma, metabolic abnormalities, or systemic symptoms that could explain his eye condition. A hypermature cataract or spontaneous rupture of the lens capsule, such as that occurring with polar cataract,^[12]^ iridogoniodysgenesis,^[13]^ Alport syndrome,^[14]^ or Marshall syndrome,^[15]^ has been reported in younger patients. Genetic factors may play an important role in some types of cataracts, such as congenital cataract, early-onset senile cataract, and systemic syndrome with cataract. At least 8 genes are known to be directly associated with age-related cataracts, including *EPHA2, GJA8*, *GALT*, *SLC16A12*, *HSF4*, *GALK1*, *FTL*, and *CRYAA*. In addition, at least 10 gene mutations can indirectly lead to cataracts, including genes that function in antioxidant metabolism (*GSTM1*, *GSTT1*), xenobiotic detoxification (*NAT2*), DNA repair (*ERCC2*), folate metabolism (*MTHFR*), lactose metabolism (*LCT*), RNA demethylation (*FTO*), lipid/cholesterol transport (*APOE4*), kinesin/microtubule motor transport (*KLC1*), and one of unknown function (*ARCC1*).^[16]^ The patient in case 1 had a hypermature cataract at a relatively young age, which is an atypical type of cataract. Whether it was complicated by genetic factors or systemic diseases requires a complete system evaluation and genetic examination. However, due to the patient’s financial constraints, we did not carry out genetic testing.

Cataract surgeries have evolved to include the development of intracapsular extraction, extracapsular extraction, and phacoemulsification. The surgical method should be individualized according to the local medical facilities, the skills of the surgeon, and the actual situation of the patient. Intracapsular cataract extraction has largely been replaced by modern cataract surgery due to many intraoperative and postoperative complications of the former procedure, including vitreous loss, cystoid macular edema, and corneal endothelial cell injury. However, intracapsular cataract extraction is still occasionally used in some underdeveloped areas.^[17]^ In these 2 cases, patients underwent intracapsular cataract extraction because of an incomplete posterior capsule. Anterior vitrectomy was performed to relieve tension on the posterior retina and prevent macular edema. Additionally, secondary intraocular lens implantation was considered to avoid severe postoperative inflammatory reactions and for accurate measurement of the intraocular lens.

In low- and middle-income countries, hypermature cataracts still account for a large proportion of cataract surgeries.^[18]^ In some remote and underdeveloped rural areas in China, patient demand for vision correction is low. Seeking treatment for the affected eye may be delayed when vision in the contralateral eye is good. Unfortunately, hypermature cataracts may cause serious complications and lead to permanent visual impairment. These adverse results can be prevented by early cataract surgery, which suggests that future work should focus on raising awareness of eye health, improving the rate of physical eye examinations, and taking effective measures to prevent the occurrence of hypermature cataracts.

## 4. Conclusion

Hypermature cataract remains a relatively common occurrence in underdeveloped areas, which can lead to many complications. Spontaneous capsular rupture with lens nucleus displacement in a hypermature lens can be seen in clinical practice. Intracapsular cataract extraction and anterior vitrectomy can easily correct this issue, and secondary intraocular lens implantation can improve vision.

## Author contributions

Conceptualization: Jie-Ying Guan, Ying-Ting Zhu and Ye-Hong Zhuo

Data curation: Yun-Cheng Ma and Aizezi Wumaier

Investigation: Jie-Ying Guan

Writing—original draft: Jie-Ying Guan

Revise and editing: Ling-Ling Xie, Mireayi Aizezi

Fund acquisition: Aizezi Wumaier
